# The RNA-Binding and RNA-Melting Activities of the Multifunctional Protein Nucleobindin 1

**DOI:** 10.3390/ijms24076193

**Published:** 2023-03-24

**Authors:** Alisa Mikhaylina, Arina Svoeglazova, Elena Stolboushkina, Svetlana Tishchenko, Olga Kostareva

**Affiliations:** Institute of Protein Research, Russian Academy of Sciences, Institutskaya, 4, 142290 Pushchino, Russia

**Keywords:** nucleobindin 1, EF-hand motif, RNA-binding activity, RNA-melting, microRNA

## Abstract

Nucleobindin 1 (NUCB1) is a ubiquitous multidomain protein that belongs to the EF-hand Ca^2+^-binding superfamily. NUCB1 interacts with Galpha_i3_ protein, cyclooxygenase, amyloid precursor protein, and lipids. It is involved in stress response and human diseases. In addition, this protein is a transcription factor that binds to the DNA E-box motif. Using surface plasmon resonance and molecular beacon approaches, we first showed the RNA binding and RNA melting activities of NUCB1. We suggest that NUCB1 could induce local changes in structured RNAs via binding to the GG$\frac{A}{U}\frac{A}{U}$ loop sequence. Our results demonstrate the importance of the multidomain structure of NUCB1 for its RNA-chaperone activity in vitro.

## 1. Introduction

Nucleobindin 1 (Calnuc, NUCB1) is a highly conserved multi-domain DNA- and calcium-binding eukaryotic protein. NUCB1 was first found in the supernatant of a B lymphocyte cell line from systemic lupus erythematosus (SLE)-prone mice [1]. In addition, systematic injections of NUCB1 into SLE-prone mice resulted in increased production of anti-dsDNA autoantibodies, anti-U1RNP antibodies, and rheumatoid factor [2]. Thus, one may assume a potential role of NUCB1 in autoimmune processes. The involvement of NUCB1 in inflammation is associated with its ability to interact with cyclooxygenase 2 [3].

Moreover, NUCB1 can act as a chaperone-like amyloid-binding protein. It was shown that NUCB1 attenuates the aggregation of unfolded proteins and protects malate dehydrogenase, alcohol dehydrogenase, and catalase under thermal stress conditions [4]. As a molecular chaperone, NUCB1 plays a protective role by interacting with the partially folded intermediates of amyloidogenic proteins. By doing this, it inhibits the aggregation of islet-amyloid polypeptide associated with type II diabetes, α-synuclein associated with Parkinson’s disease, and amyloid β peptide associated with Alzheimer’s disease by stabilizing their respective protofibril intermediates [5,6].

NUCB1 is involved in the regulation of many processes via interactions with proteins: Gαi [7], carboxyl terminus of Hsc70-interacting protein [8], APP [9], lipids [10], and DNA [11,12].

The NUCB1 gene is widely expressed in various human tissues [13], especially in cells of the endocrine system [13,14]. NUCB1 is primarily found in the endoplasmic reticulum [15], in the trans-Golgi network [14,16,17] and endosomes [15], and less frequently in the nucleus [18,19].

The protein consists of several independent functional domains: a signal peptide, a basic amino acid-rich DNA-binding domain (DBD), two Ca^2+^-binding EF-hand motifs, a C-terminal leucine zipper (Z), and a polyglutamine stretch. NUCB1 passes into various compartments of the cell and into the extracellular space due to the presence of a signal peptide [20].

EF-hand motifs of NUCB1 are very important for binding to proteins: Gαi1/3 [7], necdin [21], cyclooxygenase 2 [3], lysosome receptors [15], amyloid precursor protein (APP) [9], and the metalloproteinase MMP2 [22]. Additionally, NUCB1 may also function as a calcium sensor and a calcium buffer [23]. The NMR structure of the EF-hands domain has revealed that Ca^2+^-binding induces structural changes in the EF-hand motifs [23], which can affect NUCB1′s interdomain interactions. Presumably, Ca^2+^ ions affect NUCB1 folding [24]. It is worth noting that NUCB1 in a Ca^2+^-bound state demonstrated low chaperone activity [4].

Due to the presence of the DBD and the leucine zipper, NUCB1 possesses features of a transcription factor. It binds to the canonical enhancer box (E-box) sequence of the Cripto-1 gene promoter, triggering cell epithelial–mesenchymal transition (EMT) [12]. In addition, NUCB1 binds to DNA fragments prepared from autoimmune diseases and non-Hodgkin lymphoma cell lines [25]. In the absence of DBD or the leucine zipper, NUCB1 loses its DNA-binding properties and autoimmune response [11]. The presence of calcium ions is required for the interaction of NUCB1 with DNA [12].

Here, we report the hitherto unknown Ca^2+^-independent RNA-binding properties of NUCB1. NUCB1 was found to bind some dsRNAs and ssRNAs in vitro, including microRNAs associated with SLE and EMT. Based on our surface plasmon resonance (SPR) and chemical probing data, we suggest that NUCB1 specifically interacts with an ssRNA sequence containing guanines that may be followed by adenines or uridines. Using the molecular beacon technique, we revealed the ability of NUCB1 to melt RNA hairpin structures by means of the DBD and the leucine zipper. We suggest that NUCB1 could be involved in the regulation of translation of mRNAs associated with SLE and EMT via interaction with microRNAs.

## 2. Results

### 2.1. NUCB1 Is Able to Bind to RNA Fragments

The binding of transcription factors (TFs) to RNAs is a broad phenomenon with potentially significant biological impact [26,27]. We have studied the affinity of transcription factor NUCB1 for several single-stranded RNAs (ssRNAs) and double-stranded RNAs (dsRNAs).

First, we investigated the interaction of NUCB1 with four ssRNA fragments containing 5′-GUGGUCAGUCGAGUGG-3′ followed by adenines (A-rich), uridines (U-rich), guanines (G-rich), or cytosines (C-rich). The 5′-end RNA adapter sequence was needed for the proper orientation of the RNAs on the sensor chip. NUCB1 interacted with the A-rich, U-rich, and G-rich with high affinity. NUCB1 binding to the C-rich was not detected (Table 1, Figure S1 and File S1).

**Figure 1:**
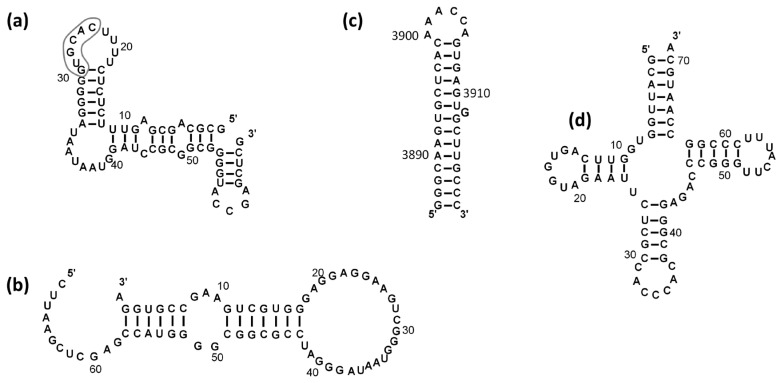
Secondary structures of (**a**) E-box RNA transcript (sequence corresponding to the E-box is outlined); (**b**) SRP19 mRNA; (**c**) Picornovirus mRNA IRES element; (**d**) Human tRNA^Gly^. The RNAfold web server (http://rna.tbi.univie.ac.at/cgi-bin/RNAWebSuite/RNAfold.cgi, accessed on 22 June 2021) was used to predict structures of the RNA fragments in (**a**–**c**).

Then, we studied the interaction of NUCB1 with dsRNAs: human tRNA^Gly^, picornavirus mRNA IRES (internal ribosome entry site) type I element, a fragment of the SRP19 mRNA 3′-UTR (SRP19), and a transcript of an E-box DNA sequence (E-box) (Figure 1). E-box RNA was chosen because NUCB1 binds to the E-box DNA sequence of a crypto-promotor [12]. The serum levels of SRP19 are changed in patients with SLE [28]. The same is true for the NUCB1 levels (our unpublished data). Thus, it is likely that NUCB1 interacts with the SRP19 mRNA 3′-UTR. It was shown that NUCB1 binds to the SRP19 mRNA and an E-box RNA with nanomolar affinity and does not interact with tRNA^Gly^ and the picornavirus mRNA IRES element (Table 1, Figure S1).

Interestingly, NUCB1 binding to RNAs did not depend on the presence of calcium ions (Table 1), which is required for the protein’s interaction with DNA [12].

Then, we checked if the NUCB-RNA interaction is structure-specific and/or sequence-specific.

### 2.2. NUCB1 Binds to the ssRNA GG$\frac{A}{U}\frac{A}{U}$ Motif

**Figure 2:**
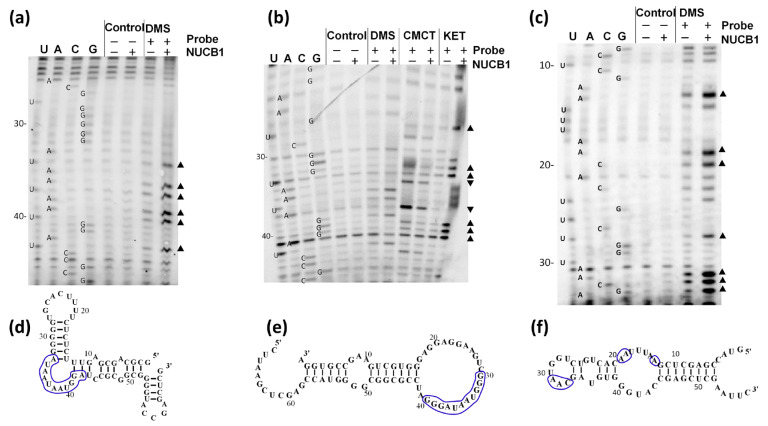
Chemical probing of the RNAs alone and in the presence of NUCB1. (**a**–**c**) Representative gels showing structure probing of E-box RNA (**a**), SRP19 mRNA (**b**), and miR-200a 3p RNA (**c**) using DMS, CMCT, and kethoxal (KET) in the presence and absence of NUCB1. Unmodified RNAs alone or in the complex with NUCB1 were used as controls. U, A, C, and G are sequencing lanes generated by reverse transcription in the presence of ddATP, ddTTP, ddGTP, and ddCTP, respectively. Nucleotides are numbered according to their position in an RNA transcript. The triangles on the right side of the gels indicate increased base reactivity upon NUCB1 addition, whereas the inverted triangles indicate the opposite. Secondary structure models of the used RNA fragments: E-box RNA (**d**), SRP19 mRNA (**e**), and miR-200a 3p RNA (**f**). Nucleotides are numbered as in (**a**–**c**). Blue lines indicate regions whose reactivity changed in the presence of NUCB1.

We studied the NUCB1/E-box RNA and NUCB1/SRP19 mRNA complexes by RNA chemical probing (Figure 2a,b).

Nucleotides, whose reactivity changes in the presence of NUCB1, are predominantly located in the single-stranded RNA regions that contain the motif GG$\frac{A}{U}\frac{A}{U}$ (Figure 2d,e). NUCB1 demonstrates a high affinity for ssRNA (Table 1). As in the single-stranded A-rich and U-rich oligo-RNAs, the A and U stretches are preceded by two guanines, and a single-stranded GG$\frac{A}{U}\frac{A}{U}$ motif is formed that can also interact with NUCB1.

NUCB1 does not bind to the hairpin of the IRES mRNA, whose loop lacks such a motif (Figure 1). It also does not bind to the highly structured tRNA^Gly^ (CCC in the anticodon loop) and the C-rich RNA. We modified the C-rich RNA sequence in order to introduce a possible NUCB1-binding motif by introducing AA after GG in the 5′-end of the C-stretch (C-rich+AA) (see Section 4). Such RNA containing the GGAA motif demonstrated high affinity for NUCB1 (K_D_ = 18 nM). Thus, one may assume that an ssRNA region containing the motif GG$\frac{A}{U}\frac{A}{U}$ is the most probable NUCB1 target.

Our recent studies suggest that NUCB1 plays a role in SLE not only in mice [29], but also in humans. It is known that single-stranded microRNAs are involved in both SLE [30,31] and EMT [32,33,34]. We assumed that the RNA-binding properties of NUCB1 apply to some microRNAs and, thus, may be physiologically important.

Interactions of NUCB1 with some microRNAs associated with the development of the SLE and EMT containing the GGAA, GGUA, GGUU, or GGAU sequences were studied. Despite the differences in the primary structures (Table 2), all these RNAs bound to NUCB1 with nanomolar affinity (Table 3).

To further dissect the NUCB1 binding site on RNA, we carried out chemical probing of the microRNA miR200a-3p NUCB1-RNA fragment in a complex with NUCB1 (Figure 2c,f). One may see that in the presence of the protein, the reactivity of the nucleotides in the “UGGUAAC” loop, including the potential NUCB1-binding motif (GG$\frac{A}{U}\frac{A}{U}$), was increased.

### 2.3. Truncated Forms of NUCB1 Bind to RNA Fragments

**Figure 3:**
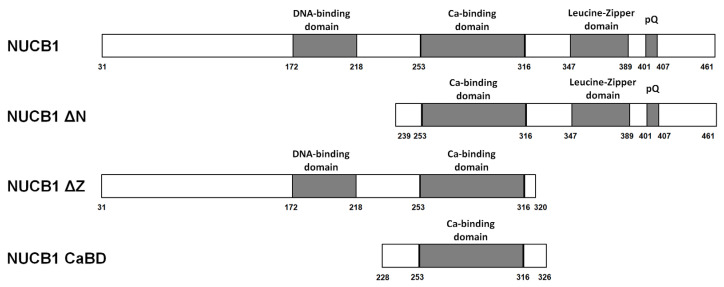
Schematic representation of NUCB1 and its truncated forms.

NUCB1 is a Ca^2+^-binding TF with an N-terminal DNA-binding domain (DBD) and a C-terminal leucine-zipper (Z) domain (Figure 3). It was established that the DBD and the Z domain are important for DNA binding [11], but their involvement in NUCB1 binding to RNA has never been shown.

The NUCB1 lacking the signal peptide (1–30 aa) was used in the following RNA-binding experiments. We obtained three truncated forms: NUCB1 ΔN (239–461 aa), with a deleted N-terminal region, including the DBD; NUCB1 ΔZ (31–320 aa), where the C-terminal region with the leucine zipper was deleted; and NUCB1 CaBD (228–326 aa), containing EF-hand motifs. The proteins were purified to homogeneity (Figure S2). The truncated forms NUCB1 ΔZ and NUCB1 CaBD were monomers, whereas NUCB1 ΔN formed dimers similarly to NUCB1 (Figure S2a).

We studied the interaction of these truncated NUCB1 variants with the RNA fragments A-rich, U-rich, C-rich, G-rich, and miR-200a-3p. Corresponding dissociation constants are listed in Table 4. Apparently, all truncated NUCB1 variants retained high affinity for RNA. Calcium ions did not affect the binding of the truncated NUCB1 forms to RNAs.

### 2.4. NUCB1 Can Melt RNA Hairpin Structures in RNAs

**Figure 4:**
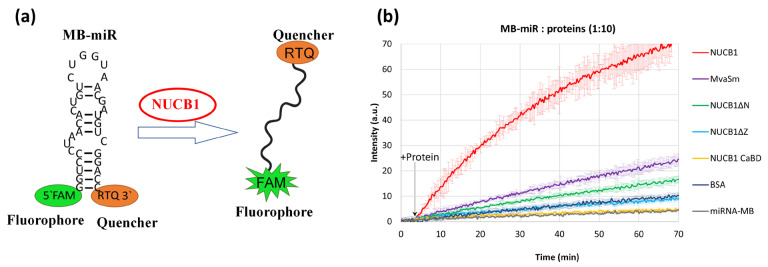
Molecular beacon melting assay. (**a**) Scheme of the MB-miR melting experiment. (**b**) Melting curves of MB-miR alone (grey curve) or after addition of proteins (colored curves). Error bars for each curve indicate the standard deviation.

Proteins that stabilize ssRNA regions can perform a chaperone function and unfold hairpin structures of RNA [35,36,37]. We suggested that NUCB1 possesses RNA chaperone activity and used the molecular beacon technique to test it [38]. The molecular beacon is a short RNA hairpin with a fluorophore at one end and a quencher at the other end of the RNA. In the hairpin state, 5′- and 3′-ends of the beacon are brought together, and the fluorescence is quenched (Figure 4a). When the RNA chaperone interacts with the molecular beacon’s loop nucleotides, the hairpin melts, the fluorophore moves away from the quencher, and fluorescence is restored.

In our experiments, a molecular beacon was used which contained the sequence of miR-200a-3p with FAM (fluorophore) at the 5′-end and RTQ (quencher) at the 3′-end of the RNA hairpin (MB-miR). Upon addition of NUCB1 to the MB-miR solution in a 1:10 molar ratio, fluorescence significantly increased in one hour (Figure 4b). The shape of the melting curve was not changed by 5-fold excess of NUCB1 over MB-miR, but the fluorescence intensity was 1.5-fold lower.

Thus, we showed that the NUCB1 was capable of unfolding hairpin structures in RNA.

For a negative control, BSA was added to MB-miR (Figure 4b). An Sm-like protein of *Methanococcus vannielii* (MvaSm), an archaeal RNA chaperone that interacts with single-stranded RNA regions containing oligoA or oligoU [37], was used as a positive control. As MB-miR does not contain a sufficient number of consecutive As or Us (more than three), the RNA chaperone activity of this protein was not pronounced.

Interestingly, the RNA-binding truncated NUCB1 variants did not exhibit RNA chaperone activity. The increase in fluorescence upon the addition of NUCB1 CaBD was indistinguishable from that of free MB-miR. The monomeric NUCB1 ΔZ, devoid of the Z domain, changed the molecular beacon fluorescence approximately as much as BSA. NUCB1 ΔN increased the fluorescence slightly more efficiently than NUCB1 ΔZ (Figure 4b).

## 3. Discussion

### 3.1. Possible Physiological Role of the RNA-Binding Properties of NUCB1

It is known that NUCB1 participates in epithelial–mesenchymal transition (EMT) via the binding to the E-box sequence of cripto promoter [12]. Presumably, the RNA-binding properties of NUCB1 could also be realized in EMT via interacting with different microRNAs. MicroRNAs are short (20–24 nt) non-coding ssRNAs that are involved in post-transcriptional regulation of gene expression by affecting both the stability and translation of mRNAs. The roles of microRNAs in EMT, SLE, and cancer are well documented [39,40,41].

The increase in serum levels of NUCB1 in lupus-prone mice became known almost 30 years ago [29]. Recently, we found elevated serum levels of NUCB1 in SLE patients as compared to those in healthy people (unpublished data). Thus, it is possible that NUCB1 could be involved not only in EMT but also in SLE via interaction with microRNAs.

We dissected NUCB1 affinity for some EMT- and/or SLE-associated microRNAs that contain a GG$\frac{A}{U}\frac{A}{U}$ motif (Table 2). Kinetic analysis has shown that these RNAs interact with NUCB1 with a nanomolar dissociation constant (Table 3). RNA chemical probing of microRNA miR-200a-3p in a complex with NUCB1 revealed that the reactivity of a number of nucleotides was increased (Figure 2c,f). This suggests that NUCB1 can stabilize single-stranded regions of physiologically important RNAs.

It is known that two populations of extracellular microRNAs exist in biological fluids, such as plasma and serum. One of them can be associated with an RNA-induced silencing complex, and the other is found in exosomes, microvesicles, and apoptotic bodies [42]. Exosomes contain different proteins, microRNAs, mRNAs, and DNA [42,43]. It was shown that exosomal NUCB1 is involved in the pathophysiology of cancer and participates in EMT and atypical migration in non-tumor cells [44]. Thus, exosomes can be a “meeting point” for microRNAs and NUCB1. We hypothesized that some microRNAs may be NUCB1 targets in exosomes.

The ability of NUCB1 to bind to some microRNAs and stabilize their single-stranded regions may shed light on how the protein is involved in various cellular processes. For instance, the overexpression of NUCB1 in neuroblastoma N2a cells is associated with reduced levels of mRNA of the amyloid precursor protein (APP). At the same time, it was shown that the microRNAs miR-106a and miR-520c repress the translation of the APP mRNA by pairing to its 3′-UTR [45]. These microRNAs contain the GG$\frac{A}{U}\frac{A}{U}$ motif in the vicinity of the regions interacting with the mRNA. NUCB1 binding to the microRNAs could stabilize their regulatory regions and/or promote pairing.

All of these factors confirm the importance of our studies of NUCB1-RNA interactions.

### 3.2. NUCB1 Binds with High Specificity to a Single-Stranded GG$\frac{A}{U}\frac{A}{U}$ RNA Sequences in a Ca^2+^-Independent Manner

NUCB1 is a multifunctional protein belonging to the family TF that binds to the E-box [12]. There are several TFs (p53, NF-YA, Sox2, Myc, TFIIIA, and YY1) that showed RNA-binding activities. Additionally, the RNA sequence that binds to transcription factors may not correspond to their specific DNA sequence [46]. For example, the TF Sox2 can interact with double-stranded regions of the non-coding RNAs ES1 and ES2, and the nucleotide sequence is not critical in this case [47]. SMAD3 binds with high affinity to RNAs containing large internal loops or bulges, and NF-kB binds to distorted RNA helices that structurally mimic DNA [48]. We showed for the first time that TF NUCB1 binds some RNA fragments (Table 1).

As we routinely used the surface plasmon resonance approach to study RNA–protein interactions for quite a long time, we obtained a number of biotinylated RNAs that specifically bound to various proteins. Initially, it was found that NUCB1 binds with high affinity to RNA fragments containing 3′-UTR of SRP19 mRNA and ssRNAs with adenines, uridines, and guanines. At the same time, NUCB1 did not bind to ssRNAs containing cytosine nucleotides, tRNA^Gly^, and picornavirus mRNA IRES element (Table 1). We further showed that the NUCB1 forms a complex with RNA encoded by E-box DNA. The affinity of NUCB1 for RNAs containing the SRP19 mRNA fragment and E-box RNA transcript, which presumably formed stem-loop structures (Figure 1), was lower than its affinity for ssRNAs (Table 1).

Additionally, we demonstrated that NUCB1 binds to RNAs in a Ca^2+^-independent manner. As it is well established that Ca^2+^ ions are required for NUCB1 binding to DNA [12], we suggest that inter-domain contacts that form in the calcium-bound form of NUCB1 [23] are not important for its binding to RNAs.

We assumed that NUCB1 could recognize a specific site in the single-stranded RNA region. A putative NUCB1-binding RNA motif was dissected using RNA chemical probing (Figure 2). SRP19 and E-box RNAs had similar regions that changed reactivity upon addition of the NUCB1 and contained the GG$\frac{A}{U}\frac{A}{U}$ motif. A similar probing pattern (increased nucleotide accessibility in the RNA–protein complex) was observed when the effect of the RNA chaperone Hfq on the conformation of the 16S rRNA was studied [49]. We suggested that NUCB1 promotes the unwinding of an RNA hairpin by binding to the loop that contains guanines, followed by adenines or uridines.

As mentioned above, NUCB1 possesses high affinity for ssRNAs containing several guanines, or the GG$\frac{A}{U}\frac{A}{U}$ motif. Two guanines were at the 3′-end of the oligo RNA, followed by oligo(A), oligo(C), or oligo(U) sequences. Interestingly, the protein did not interact with the “…GGCC…” (C-rich) sequence. However, introducing the “...GGAA…” motif to the C-rich by adding two guanines to the 3-end of the biotinylated adapter led to the formation of a strong RNA-NUCB1 complex (K_D_ = 18 nM). Thus, single-stranded GG$\frac{A}{U}\frac{A}{U}$ may serve as a NUCB1-binding motif. Furthermore, according to the results of RNA chemical probing, such interaction may lead to the unwinding of the RNA secondary structure.

### 3.3. Ca^2+^-Binding Domain of NUCB1 Is Sufficient for RNA Binding

It was shown that two regions of NUCB1 interact with DNA: the DBD, which contains a cluster of positively charged amino acid residues, and the Z domain [11,25]. At the moment, the only known spatial structure of NUCB1 is the one of the EF-hands domain [23]. The folding of the DBD and its interaction with E-box DNA were modeled based on homology to other proteins [12].

We obtained three mutant NUCB1 variants that contained the calcium-binding domain (Figure 3). This domain alone has been shown to be sufficient for RNA binding. Presumably, a portion of the EF-hands domain may overlap with the RNA-binding domains of NUCB1 [50]. The NUCB1 variant lacking the Z domain showed slightly increased affinity for RNAs in comparison with the intact protein. Similarly, to the intact NUCB1, all truncated variants bound RNAs in a calcium-independent manner.

### 3.4. NUCB1 Can Melt RNA Secondary Structures

Using the molecular beacon approach, we have shown that the NUCB1 possesses the RNA chaperone activity. This approach is widely used for studying nucleic acid chaperones. Adopting native functional conformations by cellular RNAs is a complex process that involves a number of proteins possessing an RNA chaperone activity. Such proteins destabilize intermediate secondary structures that are formed during RNA folding. By doing this, RNA chaperones promote the acquisition of thermodynamically stable conformations that are typically functionally active. RNA chaperone activity was found for a large number of proteins in various organisms. These proteins belong to evolutionary distinct protein families, and they are structurally diverse [34,51].

We synthesized a molecular beacon that contains a sequence of a potential NUCB1 target, miR-200a-3p, flanked by the 5′- and 3′-end paired extensions. The putative NUCB1-binding region is thus located within the loop structure (Figure 4a). Given that NUCB1 is an RNA chaperone, it should significantly increase fluorescence by unwinding the 5′-3′-end helix of the beacon.

The RNA chaperone MvaSm was used as a positive control because it is able to unfold hairpin RNA structures [37]. However, MvaSm bound to the A- and U-rich RNA regions, and therefore the RNA-melting activity was weakly pronounced when using MB-miR.

Adding the calcium-binding domain of NUCB1 that shows high affinity for RNA does not affect the beacon structure; the fluorescence level corresponds to that of spontaneous beacon unwinding. The truncated NUCB1 variant lacking the Z domain also virtually does not change the fluorescence. The NUCB1 variant containing the calcium-binding domain and the C-terminal part, including the Z domain, forms a dimer. Adding this variant to the beacon slightly increased the fluorescence. The intact NUCB1 increases the fluorescence intensity dramatically within several minutes of incubation, and fluorescence further increases with time. 

The spatial structure of NUCB1 is unknown. However, according to the IsUnstruct 2.02 program that predicts disordered regions in proteins [52], a significant portion of the protein, its C-terminal region in particular, is unstructured. It has been demonstrated that proteins with RNA chaperone activity had disordered regions that interact with misfolded RNAs [53,54]. These disordered regions can interact with other partners. The removal of disordered regions can lead to the loss of chaperone activity [55]. We suggest that both the N- and C-terminal regions are required for the full RNA chaperone activity of NUCB1. As mentioned above, unstructured regions are predominantly located within the C-terminus (comprising the Z domain). Thus, the C-terminus is more important for NUCB1 RNA melting activity as compared to the N-terminus.

Thus, we suggest with a high degree of confidence that NUCB1 possesses RNA chaperone activity in vitro. It is worth mentioning that both DNA-binding and RNA chaperone activity require the presence of all the domains of NUCB1. Further studies are needed to address the role of the RNA chaperone NUCB1 and find its targets in cells.

## 4. Materials and Methods

### 4.1. Production and Purification of NUCB1 and Its Truncated Forms

The open reading frame of the human NUCB1 (excluding the signal peptide) was PCR-amplified using primers the For NUCB1 and Rev NUCB1 (Table S1) and a cDNA library obtained from HEK293 T cells as a template, cloned in the pET-28a vector between EcoR I and Hind III restriction sites. The NUCB1-truncated forms (NUCB1 ΔN, NUCB1 ΔZ, and NUCB1 CaBD) genes were PCR-amplified using the primers For NUCB1 ΔN and Rev NUCB1; For NUCB1 and Rev NUCB1 ΔZ; For NUCB1 CaBD and Rev NUCB1 CaBD, respectively (Table S1), and plasmid pET-28a/NUCB1 as a template. The genes were cloned into the pET-28a vector similarly to the NUCB1 gene. The obtained genetic constructions were validated by sequencing.

The pET28a vector carrying the NUCB1 gene (or the truncated form of the NUCB1 gene) was used for transformation in *E. coli* BL21(DE3)/pRARE to express an N-terminal His6 tag protein. The transformants were grown in LB medium in the presence of kanamycin (50 µg/mL) and chloramphenicol (10 μg/mL) at 37 °C with agitation at 180 rpm. Protein expression was induced at the OD_600nm_ = 0.6–0.8 o.u. by adding IPTG to a final concentration of 0.5 mM. The bacteria were harvested by centrifugation 3 h after induction.

Cell pellets were suspended in a solution containing 500 mM NaCl, 50 mM Tris-HCl (pH 8.0), 10 mM imidazole, 5 mM β-mercaptoethanol, and 0.1 mM phenylmethylsulfonyl fluoride (PMSF). Cells were disrupted by sonication at 4 °C. Cell debris was removed by centrifugation at 15,000× *g* for 30 min at 4 °C. Supernatant was loaded onto a Ni-NTA agarose (GE Healthcare, Uppsala, Sweden) column equilibrated with a solution containing 500 mM NaCl, 50 mM Tris-HCl (pH 8.0), and 10 mM imidazole. NUCB1 variants were eluted using a step gradient of imidazole (40 mM and 150 mM) in a solution containing 500 mM NaCl and 50 mM Tris-HCl (pH 8.0). Fractions containing the protein were concentrated and dialyzed against a solution containing 150 mM NaCl and 50 mM Tris-HCl (pH 8.0). The final preparations were analyzed by size-exclusion chromatography on the Superdex 200 resin equilibrated with a solution containing 150 mM NaCl and 50 mM Tris-HCl (pH 8.0) (Figure S2).

### 4.2. Cloning and Purification of RNA Fragments

DNA fragments containing sequences of the T7 promoter and the E-box RNA, the SRP19 RNA fragment, and the miR200a-3p RNA sequences were prepared by PCR using pairs of overlapping primers: For E-box RNA and the Rev E-box RNA; For SRP19 and Rev SRP19; For miR-200a-3p and Rev miR-200a-3p, respectively (Table 2). The PCR products were inserted into vector pUC18 treated with Sma I.

The E-box, SRP19, and miR-200a-3p RNA fragments used for chemical probing experiments were obtained from linearized plasmid DNA by in vitro transcription with T7 RNA polymerase as described in [56].

### 4.3. Analysis of NUCB1-RNA Interaction

Kinetic analysis of protein interaction with specific RNA fragments was performed by surface plasmon resonance technique [57] using the ProteOn XPR36 system (Bio-Rad, Hercules, CA, USA).

The RNA-binding properties of NUCB1, the intact protein, and its truncated forms were tested using 5′-biotinylated chemically synthesized RNA oligomers (Syntol, Moscow, Russia) listed below:

U-rich: 5′-GUGGUCAGUCGAGUGG-U_18_-3′
A-rich: 5′-GUGGUCAGUCGAGUGG-A_18_-3′
C-rich: 5′-GUGGUCAGUCGAGUGG-C_6_-3′
C-rich(+AA): 5′-GUGGUCAGUCGAGUGG-AAC_6_-3′
G-rich: 5′-GUGGUCAGUCGAGUGG-G_6_-3′
miR-200a-3p: 5′-GUGGUCAGUCGAGUGGUAACACUGUCUGGUAACGAUGU-3′
miR-203a-5p: 5′-GUGGUCAGUCGAGUGGAGUGGUUCUUAACAGUUCAACAGUU-3′
miR-145-3p: 5′-GUGGUCAGUCGAGUGGGGAUUCCUGGAAAUACUGUUCU-3′
miR-155-5p: 5′-GUGGUCAGUCGAGUGGUUAAUGCUAAUCGUGAUAGGGGUU-3′
mRNA: 5′-AGGUGCCGAAGUCGUGGGAGGAGGAAGUCGGGUAAUAG-3′
E-box RNA: 5′-GCGCAGCGAGUUUCUCUCUUUUCACGUGGGGGAUAAUAAU-3′

Sixteen nucleotides (underlined) at the 5′-end of oligo RNAs and miRNAs were added to increase the accessibility of short RNA fragments for interaction with proteins. As a negative control, biotinylated tRNA^Gly^ and IRES element mRNA (Figure 1) were obtained as described [58,59,60]. Biotinylated RNA fragments were applied onto the NLC sensor chips with immobilized avidin (Bio-Rad, Hercules, CA, USA).

NUCB1 was treated with EDTA-Na_2_, followed by dialysis in a solution containing 50 mM Tris-HCl pH 8.0 and 200 mM NaCl to remove EDTA-Na_2_. Five different concentrations of the analyte samples (NUCB1 or truncated forms) were prepared by serial dilution in a solution containing 50 mM Tris-HCl (pH 8.0), 200 mM NaCl, and 0.1 % Tween-20 (TNT) for each set of sensorgrams. The samples were injected at a flow rate of 30 µL/min. The injection step included a 300–480 s association phase followed by a 2000–6000 s dissociation phase in TNT buffer. To test the effect of Ca^2+^ on the ability of NUCB1 to bind RNA, all binding experiments were performed in TNT buffer containing 2 mM CaCl_2_. All binding experiments were performed at 25 °C. Each immobilization strategy and kinetic analysis was repeated at least three times.

Kinetic analysis was performed by globally fitting curves describing the simple 1:1 bimolecular model to a set of three to five sensorgrams using BIAEvaluation v. 4.1 software.

### 4.4. RNA Chemical Probing

Structural analysis of RNAs alone (E-box, SRP19, miR-200a-3p) or in the presence of NUCB1 was performed using chemical probing of RNA as previously described [61,62,63].

#### 4.4.1. Chemical Modifications of RNA

For the chemical probing experiment, we used two types of samples: free RNAs and RNAs plus NUCB1.

RNAs were heated to 65 °C for 10 min and cooled on ice before the addition of NUCB1. Then, RNAs (3 nmoles) were mixed with NUCB1 (6 nmoles) in a buffer containing 20 mM HEPES-NaOH (pH 7.5) and 100 mM NaCl. Samples were incubated at room temperature for 30 min. Control (free RNA) samples were incubated identically.

Modification reactions (50 µL) with RNAs (750 pmoles) or RNAs with NUCB1 were performed in a buffer containing 20 mM HEPES-NaOH (pH 7.5) and 100 mM NaCl. 

Modification was performed by the addition of freshly prepared DMS (1 µL of a 1/5 dilution in ethanol), kethoxal (1 µL of a 1/5 dilution in ethanol), or CMCT (50 µL of 100 mg/mL in the reaction buffer) followed by incubation at 37 °C for 10 min. Reactions were stopped by the addition of stop-buffers, followed by ethanol precipitation. DMS stop-buffer contained 1 M Tris-HCl, pH 7.5, 1 M β-mercaptoethanol, and 100 mM EDTA-Na_2_. CMCT stop-buffer contained 3 M Na-Acetate. Kethoxal stop-buffer contained 150 mM Na-Acetate and 250 mM K_3_BO_3_ (pH 7.0).

The RNAs were concentrated by ethanol precipitation, and the pellets were washed with cold 70% ethanol. Pellets were dissolved in a buffer containing 25 mM K_3_BO_3_ (pH 7.0), 300 mM Na-Acetate, 0.5% SDS, and 5 mM EDTA-Na_2_, followed by phenol–chloroform extraction and ethanol precipitation. RNA pellets were dissolved in 10 µL of 25 mM K_3_BO_3_ (pH 7.0).

Control (unmodified) samples were treated in the same way as modified samples, except that the modification step was skipped.

Unmodified RNAs were used as templates for the reference sequencing reactions and to monitor artifact stops or pauses in reverse transcription.

#### 4.4.2. Fluorescent Primer Extension

RNA fragments used in chemical probing experiments contain identical 3′-end, which correspond to the pUC18 sequence between restriction sites Sma I and EcoR I (5′-GGGUACCGAGCUCGAAUUC-3′). A Cy5 fluorescent reverse transcription primer complementary to the 3′-end of the studied RNA fragments was purchased from Evrogen (Moscow, Russia).

For reverse transcription (RT), RNAs or modified RNAs (4 µL of a 1 µM solution) were added to the Cy5-primer (4 µL of a 2 µM solution) in an annealing buffer (250 mM Tris-HCl (pH 8.3), 200 mM KCl), heated to 90 °C for 3 min, and slowly cooled to 37 °C. RT reactions were performed in 50 mM Tris-HCl (pH 8.3), 40 mM KCl, 6 mM MgCl_2_, and 2 mM each dNTP. M-MuLV Reverse Transcriptase 5 units (SibEnzyme, Nowosibirsk, Russia) were added, and reactions were incubated at 37 °C for 60 min. For sequencing samples, 1 µM ddATP, ddCTP, ddGTP, or ddTTP were added to the RT reactions of each sample. Reactions were stopped by adding formamide with 10 mM EDTA, 0.3% bromophenol blue, and 0.3% xylene cyanol. Aliquotes of 6–8 µL were analyzed in an 8% polyacrylamide gel in the presence of 8 M Urea. Gels were visualized using the ChemiDoc MP Imaging System (BioRad, Hercules, CA, USA).

### 4.5. Molecular Beacon Melting Assay

A Cary Eclipse fluorescence spectrometer (Varian, Palo Alto, CA, USA) was used in the assay. All measurements were performed in a 0.3×0.3 cm cuvette at a constant temperature of 22 °C; the excitation wavelength was 496 nm, and emission wavelength was 519 nm.

To study the RNA chaperone properties of NUCB1 protein, chemically synthesized molecular beacon-microRNA-200a-3p (MB-miR) were used (Syntol, Moscow, Russia). A MB-miR is a hairpin RNA containing the microRNA-200a-3p RNA sequence in a loop with a FAM fluorophore at one end and an RTQ1 quencher (analog of BHQ1) at the other end (Figure 4a). In the initial state, the 5′- and 3′- ends of MB-miR are in close proximity, so the fluorescence is quenched.

Analysis of protein-induced changes in fluorescence intensity was performed for NUCB1, its truncated forms, MvaSmAP1 as a positive control, and BSA as a negative control.

Proteins were added at 1:5 and 1:10 molar ratios to 200 nM MB-miR in 100 µL of 10 mM Tris-HCl (pH 8.0) and 100 mM NaCl. Three independent melting curves were obtained for each MB-miR-protein combination.

## 5. Conclusions

In this study, we have shown for first time that NUCB1 possesses RNA-binding and RNA-melting activities. Apparently, the protein can bind to the ssRNA GG$\frac{A}{U}\frac{A}{U}\;$ motif and trap RNA in a kinetically favored conformation. Although the calcium-binding domain of NUCB1 alone is sufficient for the RNA binding, efficient RNA-melting function requires the presence of all NUCB1 domains.

## Figures and Tables

**Table 1:** Kinetic constants and equilibrium dissociation constants (K_D_) of the NUCB1-RNA complexes in the presence or absence (*) of Ca^2+^ ions.

RNA	k_a__(M−1·s−1)_	k_d__(s−1)_	K_D__(nM)_
IRES			Not detected
tRNA^Gly^			Not detected
C-rich			Not detected
U-rich	2.9 × 10^5^	3.2 × 10^−4^	1.1 ± 0.1
	1.9 × 10^5^ *	3.6 × 10^−4^ *	1.9 ± 0.3 *
A-rich	2.9 × 10^5^	3.4 × 10^−4^	1.2 ± 0.1
	2.1 × 10^5^ *	2.9 × 10^−4^ *	1.4 ± 0.1 *
G-rich	3.4 × 10^5^	5.1 × 10^−4^	1.5 ± 0.2
	3.8 × 10^5^ *	4.5 × 10^−4^ *	1.2 ± 0.3 *
SRP19	1.3 × 10^5^	1.7 × 10^−3^	13.2 ± 0.6
	2.5 × 10^5^ *	4.3 × 10^−3^ *	17.2 ± 0.5 *
E-box	5.8 × 10^5^	6.7 × 10^−3^	11.4 ± 0.8
	8.2 × 10^5^ *	8.4 × 10^−3^ *	10.2 ± 0.7 *

**Table 2:** Nucleotide sequences of microRNAs that were used for SPR studies. The potential NUCB1-binding motif (GG$\frac{A}{U}\frac{A}{U}$ ) is underline.

microRNA	Sequence
miR-200a-3p	5′-UAACACUGUCUGGUAACGAUGU-3′
miR-203a-5p	5′-AGUGGUUCUUAACAGUUCAACAGUU-3′
miR-145-3p	5′-GGAUUCCUGGAAAUACUGUUCU-3′
miR-155-5p	5′-UUAAUGCUAAUCGUGAUAGGGGUU-3′

**Table 3:** Kinetic analysis of the interaction of NUCB1 with microRNA.

RNA	k_a__(M−1·s−1)_	k_d__(s−1)_	K_D__(nM)_
miR-145-3p	5.4 × 103	2.6·10−5	4.9 ± 0.2
miR-155-5p	2.2 × 103	3.6·10−6	1.7 ± 0.1
miR-200a-3p	1.9 × 104	7.4·10−5	3.8 ± 0.1
miR-203a-5p	3.5 × 104	3.2·10−4	9.3 ± 0.4

**Table 4:** The K_D_ (nM) of complexes formed by the NUCB1 variants with RNAs.

RNA	Truncated NUCB1 Variants		
	∆N	∆Z	CaBD
miR-200a-3p	0.6 ± 0.09	0.3 ± 0.01	5.2 ± 0.2
C-rich	Not detected	Not detected	Not detected
A-rich	1.4 ± 0.1	0.3 ± 0.02	5.7 ± 0.5
U-rich	1.7 ± 0.3	0.4 ± 0.03	0.2 ± 0.04
G-rich	5.3 ± 0.6	3.1 ± 0.2	18.2 ± 3

## Data Availability

Not applicable.

## Supplementary Materials

The following supporting information can be downloaded at: https://www.mdpi.com/article/10.3390/ijms24076193/s1.
