# Supplementary material for: The RNA-Binding and RNA-Melting Activities of the Multifunctional Protein Nucleobindin 1
Source: Int J Mol Sci. 2023 Mar 24;24(7):6193. doi: 10.3390/ijms24076193 (PMC10093973; doi:10.3390/ijms24076193)
Supplement: Supplementary file 1 [file ijms-24-06193-s001.zip › ijms-2281685-supplementary.pdf]

Supplementary materials

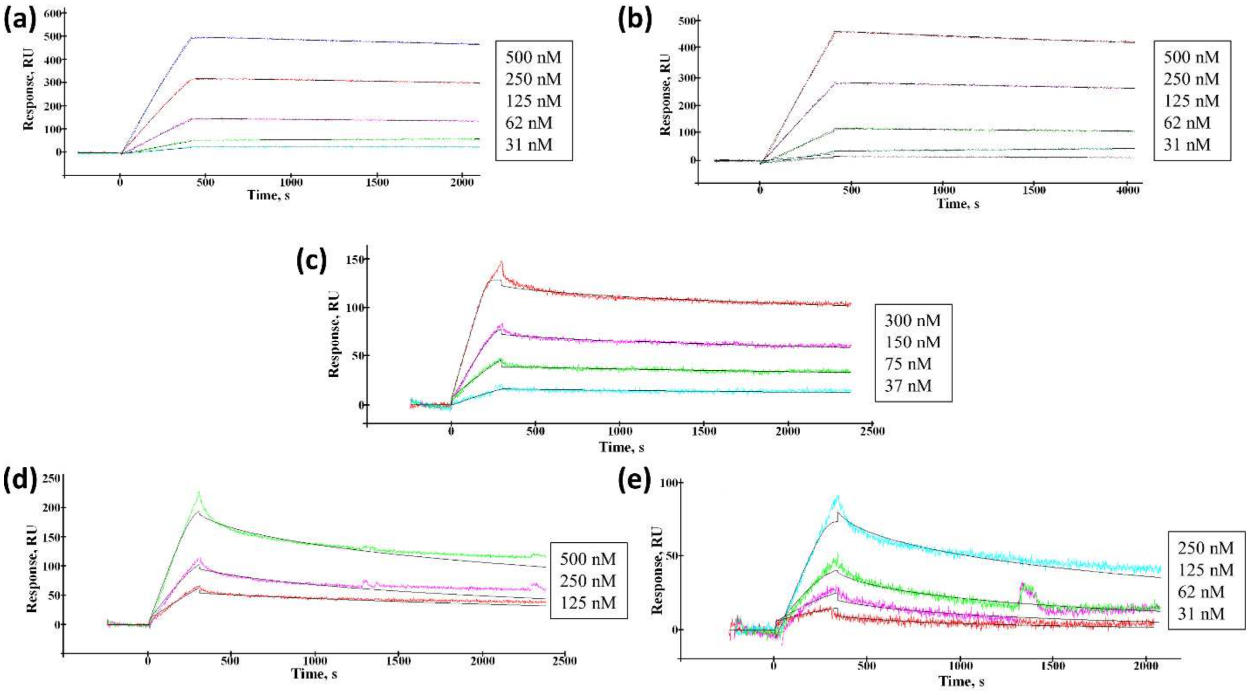

Figure S1. Sensograms showing binding kinetics for the NUCB1-RNA fragments: (a) NUCB1 with U-rich RNA, (b) NUCB1 with A-rich RNA, (c) NUCB1 with G-rich RNA, (d) NUCB1 with SRP19 mRNA, (e) NUCB1 with E-box RNA. The analyte concentrations used for each data set are shown. Grey lines represent the global fit of the data sets using a 1:1 model.

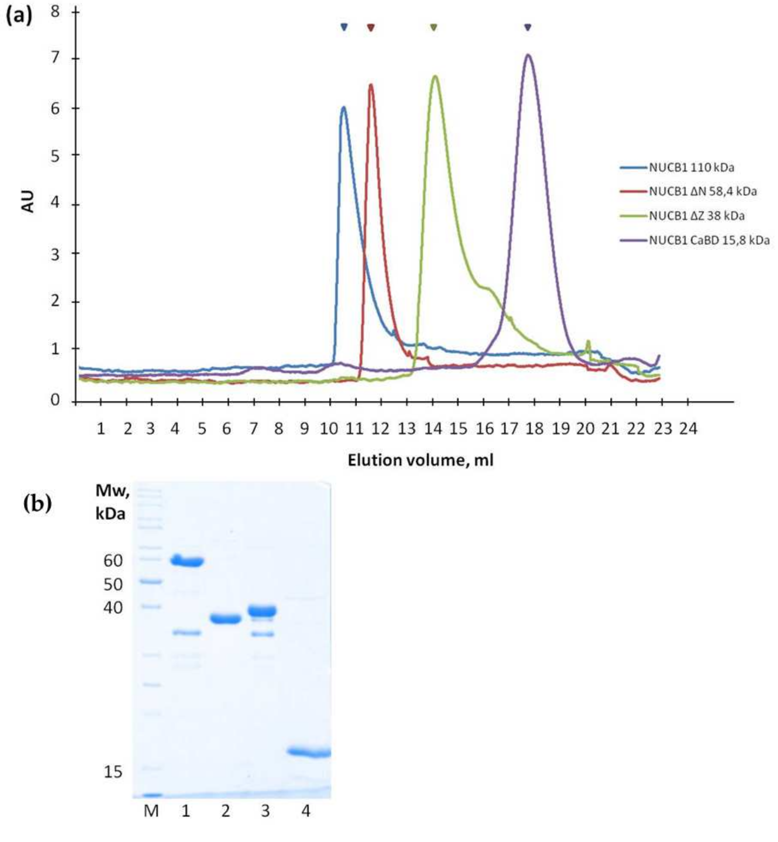

Figure S2. Purification of NUCB1 and its truncated forms. (a) Elution profiles of size exclusion chromatography on the HiLoad Superdex 200 16/60 column of NUCB1 and its truncated forms. The centers

of elution peaks indicate by triangles, molecular weights of proteins are indicated next to the names. (b) Analyzing protein preparations by SDS-PAGE. Lane M – protein size marker, lane 1 – NUCB1, lane 2 - NUCB1 ΔN, lane 3 - NUCB1 ΔZ, lane 4 - NUCB1 CaBD.

Table.S1. Primers used in this study.

Name	Sequence, 5'→3'
For NUCB1	GCGGATCCGAATTCCGAGGGGCGCCCAACAAGGAGGAG
Rev NUCB1	GCGGCCGCAAGCTTTCACAGATGCTGGGGCACCTC
For NUCB1 ΔN	GCGGATCCGAATTCGACCCCAACAGGTTTAACCCCAAG
Rev NUCB1 ΔZ	CACCCCAAGCTTTTAGGATGCGAGGAACTCCTCCAG
For NUCB1 CaBD	ATTATCCGAATTCTTGAAGGAGGTGTGGGAGGAGCTG
Rev NUCB1 CaBD	CACCCCAAGCTTTTATCAAAACTCCTTCCTCTGAGTGG
For E-box RNA	TAATACGACTCACTATAGGGCGCAGCGAGTTTCTCTCTTTTC
Rev E-box RNA	GGATCCATTATTATCCCCCACGTGAAAAGAGAGAAAC
For SRP19	TAATACGACTCACTATAGGAGGTGCCGAAGTCGTGGGAGG
Rev SRP19	CGCCGCGGATCCCTATTACCCGACTTCCTCCTCCCACGACTTC
For miR-200a-3p	TAATACGACTCACTATAGGGTACCGAGCTCGAATTT
Rev miR-200a-3p	ACATCGTTACCAGACAGTGTTAAATTCGAGCTCGGTACCC
Cy5-primer	Cy5-GAATTCGAGCTCGGTACCC
